# Illegal Drug Use and Risk of Hearing Loss in the United States: A National Health and Nutrition Examination Survey

**DOI:** 10.3390/ijerph182211945

**Published:** 2021-11-13

**Authors:** Po-Ting Lin, I-Hsun Li, Hui-Wen Yang, Kuan-Wei Chiang, Chih-Hung Wang, Li-Ting Kao

**Affiliations:** 1 School of Pharmacy, National Defense Medical Center, Taipei 11490, Taiwan; ilikehei26@gmail.com (P.-T.L.); lhs01077@gmail.com (I.-H.L.); 2 Department of Pharmacy Practice, Tri-Service General Hospital, Taipei 11490, Taiwan; 3 Department of Pharmacology, National Defense Medical Center, Taipei 11490, Taiwan; 4 Graduate Institute of Life Sciences, National Defense Medical Center, Taipei 11490, Taiwan; e0936061277@gmail.com; 5 Department of Otolaryngology-Head and Neck Surgery, Taipei Veterans General Hospital, Taipei 11217, Taiwan; weiskyli@gmail.com; 6 Department of Otorhinolaryngology-Head and Neck Surgery, Tri-Service General Hospital, Taipei 11490, Taiwan; 7 School of Public Health, National Defense Medical Center, Taipei 11490, Taiwan

**Keywords:** illegal drug, hearing loss, national health and nutrition examination survey

## Abstract

The use of illegal drugs may be a risk factor of hearing loss. However, very few studies with large sample size have investigated the relationship between illegal drug use and hearing loss. Therefore, to evaluate the association between illegal drug use and hearing loss, this cross-sectional population-based study collected data from the US National Health and Nutrition Examination Survey 2011. The study included 1772 participants aged 20 to 59 years who underwent the Drug Use Questionnaire and Audiometry Examination. Of the 1772 participants in this study, 865 were men (48.8%) and 497 were illegal drug users. The mean (SD) age of the patients was 40.0 (11.4) years. After considering age, sex, and comorbidities, the participants who used illegal drugs were found to have higher risks of high-frequency hearing loss (adjusted odds ratio (OR), 1.69; 95% confidence interval (CI), 1.35–2.10) and overall hearing loss (adjusted OR, 1.69; 95% CI, 1.36–2.12) as compared with the nonusers. In the second analysis, the participants who used ≥ 2 types of illegal drugs were associated with higher risks of high-frequency hearing loss (adjusted OR, 1.57; 95% CI, 1.06–2.32) and overall hearing loss (adjusted OR, 1.60; 95% CI, 1.08–2.37). In the third analysis, cocaine use was associated with increased risks of high-frequency hearing loss (adjusted OR, 1.34; 95% CI, 1.01–1.77) and overall hearing loss (adjusted OR, 1.38; 95% CI, 1.04–1.82). The adjusted OR for overall hearing loss in the methamphetamine users was 1.54 (95% CI, 1.05–2.27) as compared with that in the nonusers. This study shows that illegal drug users might have a higher risk of overall hearing loss than nonusers. In addition, the analysis results demonstrated that the more kinds of illegal drugs used, the higher the risk of hearing loss. Further experimental and longitudinal research studies are required to confirm the causal relationship between illegal drug use and hearing loss.

## 1. Introduction

Hearing loss is a common chronic disorder and affects approximately 466 million people worldwide [1,2]. In addition to functional problems, hearing loss may contribute to social, vocational, and mental problems [3,4]. For instance, poor auditory sensitivity may result in communication interruptions, and inefficient listening environments commonly lead to more accidents [5]. Some people with hearing loss also have lower wages and higher unemployment rates [6]. Furthermore, some recent studies demonstrated the associations between hearing loss, depression, cognitive decline, and dementia [7]. Even though hearing loss is recognized as an important public health issue, to date, the actual risk factors and etiologies are still controversial [8]. Many clinicians consider hearing loss to be possibly caused by genetic problems, ear infections, drug side effects, diseases, age, and excessive noise [2]. Recent studies have indicated that the use of some substances may be a risk factor of hearing loss [9].

Illegal drug use is recognized as a major public concern and may contribute to health and social problems [10,11,12]. In 2018, approximately 269 million people worldwide had used drugs at least once in the previous year [13]. Of these people, 35.6 million developed drug use-related disorders [13]. Furthermore, 42 million years of “healthy” life lost and 585,000 deaths were attributed to the use of drugs [13]. This situation poses a threat to personal health and family stability. Therefore, estimation of the potential effects of illegal drug use is essential. Many previous studies found that illegal drugs may affect the nervous and cardiovascular systems, so people using drugs have higher risks of developing central nervous system (CNS) diseases, cardiovascular diseases, and mental disorders [14]. In addition, some experimental studies found that illegal drugs have an impact on neurotransmitters [15,16,17]. Accordingly, the abnormality of neurotransmitters may be associated with hearing loss [16,17]. An increasing number of case reports also illustrated that the use of illegal drugs is associated with hearing loss [18,19,20].

However, although illegal drugs and hearing loss are all major public concerns, to date, the existing population-based research studies with large sample sizes that investigated the association between the use of illegal drugs and hearing loss are still insufficient. Owing to the ethical issue and difficulties in recruiting participants, most previous studies were only basic research and case reports. Therefore, this study aimed to use large-scale data from the US National Health and Nutrition Examination Survey (NHANES) to determine the actual association between illegal drug use and hearing loss.

## 2. Materials and Methods

### 2.1. Database

The data used in this cross-sectional study were sourced from the US NHANES. NHANES has been a continuous program of the National Center for Health Statistics (NCHS) for health and nutrition measurements since 1999. The database includes interview and physical examination data. The sample of approximately 5000 persons from counties across the United States that are included in the yearly NHANES investigation is nationally representative. The NHANES research protocols were approved by the research ethics review board of the NCHS, and all the participants provided their written informed consent to the NCHS. These survey data are free, publicly available, and can be directly downloaded on the NHANES website (https://www.cdc.gov/nchs/nhanes/index.htm) (accessed on 12 November 2021) for data users and researchers throughout the world. As the publicly available database did not include the participants’ identifying information, our study was exempt from a full review by the institutional review board.

### 2.2. Study Sample Selection

**Figure 1:**
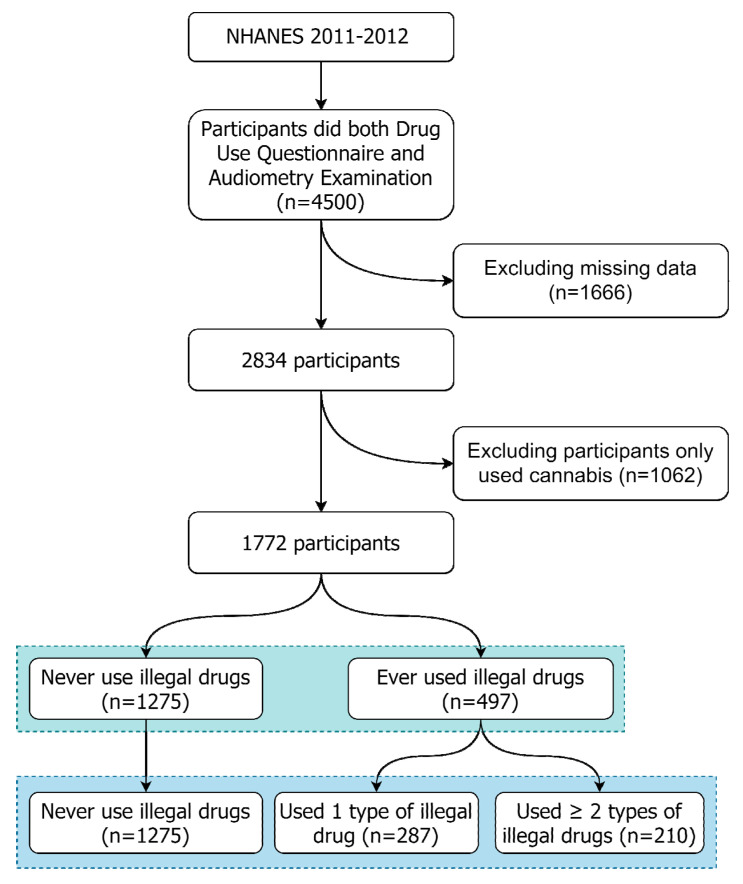
Flow diagram for the selection of sampled patients.

This study first included respondents from ages 20 to 69 years who had received both the Drug Use Questionnaire and Audiometry Examination during the period of 2011–2012 (*n* = 4500). All missing data in the questionnaires and examinations were excluded (*n* = 1666). We further excluded respondents who only used cannabis as indicated in the Drug Use Questionnaire (*n* = 1062) because cannabis is legal in several counties. Finally, 497 participants were defined as illegal drug users for inclusion as the exposed group, and 1275 participants were defined as nonusers for inclusion as the control group. Overall, 1772 participants were included in the final analyses. Illegal drug users were identified by using the following questions from the Drug Use Questionnaire: “Ever use any form of cocaine?” “Ever used heroin?,” and “Ever used methamphetamine?”. Participants who responded “yes” to any of the three previous questions were identified as illegal drug users in this study. Flow diagram for the selection of sampled patients was displayed in Figure 1. In addition, the definitions of illicit drug use and hearing loss have been assured in previous studies [21,22].

### 2.3. Outcome Measurement

This study attempted to investigate the association between illegal drug use and hearing loss. The definitions of hearing loss were based on the audiometry examination results. All the selected participants in this study were examined for pure tone air-conduction thresholds of each ear with standardized audiometry protocols and equipment. The frequency of the pure tone air-conduction threshold test ranged from 0.5 to 8 kHz, and the intensity range of the test was from −10 to 120 dB. We used Shargorodsky’s definitions of hearing loss as follows [23]: low-frequency hearing loss was identified as the pure tone average at 0.5, 1, and 2 kHz thresholds ≥ 15 dB. High-frequency hearing loss was defined as the pure tone average at 3, 4, 6, and 8 kHz thresholds ≥ 15 dB. Both of the two previous types of hearing loss were defined as overall hearing loss.

### 2.4. Covariate Measurement

Hearing loss was associated with age, sex, race, hypertension, ear infection, and diabetes [22,23]. Thus, we included the previous variables as potential confounders. Age was used as a continuous variable. Sex was classified as male or female. Race was classified into five categories, namely, Mexican American, other Hispanic, non-Hispanic White, non-Hispanic Black, and other races (including multi-racial). Ear infection and hypertension statuses were assessed using self-report questionnaires. The laboratory data of glycohemoglobin were used to classify diabetes status into diabetics and non-diabetics.

### 2.5. Statistical Analyses

All analyses in this study used the SPSS system (version 22, IBM Corp., Armonk, New York, NY, USA). We compared the exposed and control groups in terms of demographic characteristics and comorbidities by using the chi-square test, independent t test, mean, and standard deviation. We further used logistic regression models to evaluate the association between the two groups and calculated unadjusted and adjusted odds ratios (ORs) and 95% confidence intervals (CIs). CI was defined as a range of values in which the true parameter was expected to fall. Additionally, age, gender, race, hypertension, ear infection, and diabetes were all adjusted in the logistic regression models. In all the statistical analyses, *p* values < 0.05 indicated a statistically significant difference.

## 3. Results

### 3.1. The Demographics and Comorbidities of the Selected Participants

This cross-sectional study consisted of 1772 people aged ≥ 20 years. The mean age of all the participants was 40.0 ± 11.4 years. Table 1 displays the demographics and comorbidities of the selected participants in this study. In the full cohort, the results showed significant differences between the illegal drug users and nonusers in terms of age (*p* = 0.012), sex (*p* < 0.001), race (*p* < 0.001), hypertension (*p* < 0.001), and ear infection (*p* < 0.001), but not in terms of the incidence of diabetes (*p* = 0.954). This study further classified the illegal drug users into two groups as follows: (1) those who were using 1 type of illegal drug and (2) those who were using ≥ 2 types of illegal drugs. The relevant findings presented significant differences between the groups (users of 1 type of illegal drug vs. users of ≥2 types of illegal drugs vs. nonusers) in terms of age (*p* < 0.001), sex (*p* < 0.001), race (*p* < 0.001), hypertension (*p* < 0.001), and ear infection (*p* < 0.001), but not in terms of the incidence of diabetes (*p* = 0.917). We considered these factors in the following regression models.

This cross-sectional study consisted of 1772 people aged ≥ 20 years. The mean age of all the participants was 40.0 ± 11.4 years. Table 1 displays the demographics and comorbidities of the selected participants in this study. In the full cohort, the results showed significant differences between the illegal drug users and nonusers in terms of age (*p* = 0.012), sex (*p* < 0.001), race (*p* < 0.001), hypertension (*p* < 0.001), and ear infection (*p* < 0.001), but not in terms of the incidence of diabetes (*p* = 0.954). This study further classified the illegal drug users into two groups as follows: (1) those who were using 1 type of illegal drug and (2) those who were using ≥ 2 types of illegal drugs. The relevant findings presented significant differences between the groups (users of 1 type of illegal drug vs. users of ≥2 types of illegal drugs vs. nonusers) in terms of age (*p* < 0.001), sex (*p* < 0.001), race (*p* < 0.001), hypertension (*p* < 0.001), and ear infection (*p* < 0.001), but not in terms of the incidence of diabetes (*p* = 0.917). We considered these factors in the following regression models.

### 3.2. Prevalence, Odds Ratios, and 95% Confidence

Table 2 shows the prevalence of hearing loss in the sampled participants, and we analyzed the association between illegal drug use and hearing loss. High-frequency hearing loss was found in 341 illegal drug users (68.8%) and 720 nonusers (56.5%). In addition, overall hearing loss was found in 348 illegal drug users (70%) and 739 nonusers (58%). The univariate logistic regressions revealed that the participants who used illegal drugs had higher risks of high-frequency hearing loss (OR, 1.69; 95% CI, 1.35–2.10) and overall hearing loss (OR, 1.69; 95% CI, 1.36–2.12) than the nonusers. To eliminate the potential bias in this study, the participants’ demographics and comorbidities were all considered in the regression models. After adjustments, we found that the illegal drug users had higher risks of high-frequency hearing loss (OR, 1.32; 95% CI, 1.00–1.73) and overall hearing loss (OR, 1.38; 95% CI, 1.05–1.82) than the nonusers.

Table 3 further displays the prevalence of hearing loss and the association between the number of illegal drugs used and hearing loss. Relevant findings indicated that the risk of high-frequency hearing loss was higher in the users of 1 type of illegal drugs (OR, 1.51; 95% CI, 1.15–1.98) and users of ≥ 2 types of illegal drugs (OR, 1.97; 95% CI, 1.43–2.72) than in the nonusers. In addition, compared with the nonusers, a higher risk of overall hearing loss was found in the users of 1 type of illegal drugs (OR, 1.54; 95% CI, 1.17–2.02) and users of ≥2 types of illegal drugs (OR, 1.95; 95% CI, 1.41–2.69). After considering confounders, the adjusted OR of high-frequency hearing loss was 1.57 (95% CI, 1.06–2.32) for the users of ≥2 types of illegal drugs as compared with nonusers, and the adjusted OR of overall hearing loss was 1.60 (95% CI, 1.08–2.37) for the users of ≥ 2 types of illegal drugs as compared with the nonusers. Accordingly, illegal drug use may increase the risks of high-frequency and overall hearing loss. The participants who used ≥ 2 types of illegal drugs had the highest risk of high-frequency and overall hearing loss.

Table 4 presents the relationship between the respective uses of different illegal drugs and hearing loss. After adjusting for the participants’ age, sex, race, hypertension, ear infection, and diabetes, the adjusted OR of high-frequency hearing loss was 1.34 (95% CI, 1.01–1.77) for cocaine users as compared with nonusers, and the adjusted OR of overall hearing loss was 1.38 (95% CI, 1.04–1.82) for cocaine users as compared with nonusers. The adjusted OR of overall hearing loss was 1.54 (95% CI, 1.05–2.27) for methamphetamine users as compared with nonusers.

## 4. Discussion

This study used the US NHANES data and found that illegal drug users had higher odds of developing overall and high-frequency hearing loss than nonusers even after adjustment for their demographic characteristics and comorbidities. This study further demonstrated that the more kinds of illegal drugs used, the higher the risk of hearing loss. In addition, the relevant findings include the possible association of cocaine use with high-frequency and overall hearing loss. Methamphetamine use may also be related with overall hearing loss after considering confounders.

To date, only a few population-based studies with large sample sizes have investigated the relationship between illegal drug use and hearing loss. Most previous human studies only attempted to demonstrate the relationship between alcohol and cigarette use and hearing loss [24,25,26,27]. A cross-sectional study with a large sample size in the United States further observed that hearing loss may be associated with substance use-related disorders (especially alcohol and prescription opioid use) among subjects aged ≤ 49 years [9]. Our study provides complete information about the connection between illegal drug use and hearing loss, and the findings in this study are consistent with the consequences of using illegal drugs in prior case reports and small trials [19,20,28,29,30]. For instance, a woman attended a clinic for a profound bilateral hearing loss after an intravenous injection of cocaine [19]. Pure tone audiometry revealed a severe symmetrical sensorineural hearing loss. However, she was not on any other medication and was in reasonably good health, with no significant past medical history [19]. In addition, a 31-year-old man developed a profound sensorineural hearing loss after using heroin. The audiogram demonstrated a moderate-to-severe bilateral sensorineural hearing loss [28].

Although the exact mechanisms of hearing impairment and the association between the use of illegal drugs and hearing loss are hypothetical and unclear, there are two probable reasons regarding hearing loss and its types. First, it is plausible that neurotransmitters may play roles in the association between illegal drug use and hearing. To date, many studies have found that neurotransmitters may affect audition [15,17,31]. An experimental study used electrophysiological recordings to evaluate the effects of dopamine in mice’s midbrain nuclei [31]. In another study, the investigators demonstrated that serotonin shifts the representation of convergent auditory and multisensory pathways in mice’s dorsal cochlear nuclei [17]. Moreover, the illegal drugs we investigated in this study are CNS stimulants. Cocaine and methamphetamine have been recognized to increase the concentrations of dopamine, serotonin, and norepinephrine [15]. Heroin activates the receptors of opioids [32]. These relationships may explain the association found in this study because most illegal drugs and auditory functions may share similar biological mechanisms.

Second, the types of hearing loss are related to lesions of different cochlear sites. We found that the type of hearing loss more frequently associated with illegal drug use was high-frequency hearing loss. In this study, the participants who used illegal drugs had a higher risk of high-frequency hearing loss than the nonusers (adjusted OR, 1.32). A higher risk of high-frequency hearing loss was found in both the users of 1 type of illegal drug (adjusted OR, 1.18) and users of ≥2 types of illegal drugs (adjusted OR, 1.57) than in the nonusers. Cocaine use was also observed to be associated with high-frequency hearing loss. The pure tone audiograms of the previous case reports also showed similar trends [20,28]. For instance, a man developed severe hearing loss after intravenous injection of cocaine [20]. The audiogram on admission showed symmetrical air-conduction levels of up to 80 dB at 4 kHz, which was defined as high-frequency in the audiometry [20]. However, the actual causes of high- and low-frequency hearing loss are unclear. One previous study indicated that each cochlear site has a characteristic frequency to which it responds maximally [33]. Thus, we could presume that the illegal drugs would affect a specific cochlear site and contribute to the development of high-frequency hearing loss. However, more studies are still required to determine the mechanisms of this association. This study has some strengths. First, it used a large, nationally representative survey in the United States, which provided a sufficient sample and statistical power. Second, this study used pure tone audiometry results to determine the participants’ auditory statuses and classified hearing loss into two types (low- and high-frequency hearing loss) according to the pure tone audiometric frequency. This examination was much more accurate than the self-reported hearing loss in many prior research studies. The information about low- and high-frequency hearing loss could provide more clinical evidence to physicians. Third, this study focused on the effects of illegal drug use. Furthermore, the relevant analyses investigated the relationship between illegal drug use and hearing loss according to the number of illegal drugs used and type of illegal drug use. Owing to some ethical issues and design difficulty, the relevant records used in this study were valuable and could provide detailed information to clinicians.

This study has some limitations. First, the study focused on illegal drugs, including cocaine, heroin, and methamphetamine. As this study used questionnaires to identify illegal drug users, the number of illegal drug users might have been underestimated. Second, the self-reported questionnaire may cause information bias. In addition, recall bias may be caused by the participants’ incorrect recall of using illegal drugs. Third, owing to the cross-sectional design of the study, we could not determine the causal relationship between hearing loss and the use of illegal drugs.

## 5. Conclusions

In conclusion, this study demonstrated the association between the use of illegal drugs and hearing loss, including high-frequency and overall hearing loss. In addition, we found that the risk of overall hearing loss for the users of ≥2 types of illegal drugs was high. Our study also revealed that cocaine and methamphetamine users had higher risks of hearing loss than the nonusers. The relevant information could provide some evidence for policy makers and medical professionals. However, further experimental and longitudinal research studies are required to confirm the causal relationship between illegal drug use and hearing loss.

## Figures and Tables

**Table 1:** Demographic characteristics and comorbidities of selected participants.

Characteristic		Full Cohort					Numbers of Illegal Drug Use						
		Illegal Drug Users (*n* = 497)		Nonusers (*n* = 1275)		*p* Value	Used 1 Type of Illegal Drug (*n =* 287)		Used ≥ 2 Types of Illegal Drugs (*n* = 210)		Nonusers (*n* = 1275)		*p* Value
		No.	%	No.	%		No.	%	No.	%	No.	%	
Age (Mean ± SD)		41.9 ± 10.7		39.2 ± 11.6		0.012	42.0 ± 11.0		41.8 ± 10.2		39.2 ± 11.6		<0.001
Sex						<0.001							<0.001
	Male	301	60.6	564	44.2		169	58.9	132	62.9	564	44.2	
	Female	196	39.4	711	55.8		118	41.1	78	37.1	711	55.8	
Race						<0.001							<0.001
	Mexican American	57	11.5	163	12.8		32	11.1	25	11.9	163	12.8	
	Other Hispanic	33	6.6	146	11.5		24	8.4	9	4.3	146	11.5	
	Non-Hispanic White	267	53.7	345	27.1		134	46.7	133	63.3	345	27.1	
	Non-Hispanic Black	91	18.3	312	24.5		66	23	25	11.9	312	24.5	
	Other race	49	9.9	309	24.2		31	10.8	18	8.6	309	24.2	
Hypertension ^a^						<0.001							<0.001
	Yes	149	30	276	21.7		74	25.9	75	35.7	276	21.7	
	No	347	70	998	78.3		212	74.1	135	64.3	998	78.3	
Ear infection ^a^						<0.001							<0.001
	Yes	142	29.2	237	19.1		79	28.2	63	30.6	237	19.1	
	No	344	70.8	1001	80.9		201	71.8	143	69.4	1001	80.9	
Diabetes ^a^						0.954							0.917
	Yes	36	7.5	93	7.6		22	7.9	14	6.9	93	7.6	
	No	443	92.5	1131	92.4		255	92.1	188	93.1	1131	92.4	

Note: ^a^ The numbers (No.) do not add up to the total recruited number because of the missing data.

**Table 2:** Prevalence, odds ratios, and 95% confidence interval for low-frequency hearing loss, high-frequency hearing loss, and hearing loss, stratified by whether or not patients used illegal drugs.

Variables		Illegal Drug Users (*n* = 497)		Nonusers (*n* = 1275)	
		No.	%	No.	%
Low-Frequency Hearing Loss					
	Yes	138	27.8	324	25.4
	No	359	72.2	951	74.6
	Crude OR (95% CI) ^a^	1.13 (0.89–1.43)		Ref.	
	Adjusted OR (95% CI) ^a,b^	0.90 (0.69–1.19)		Ref.	
High-Frequency Hearing Loss					
	Yes	341	68.6	720	56.5
	No	156	31.4	555	43.5
	Crude OR (95% CI) ^a^	1.69 *** (1.35–2.10)		Ref.	
	Adjusted OR (95% CI) ^a,b^	1.32 * (1.00–1.73)		Ref.	
Overall Hearing Loss					
	Yes	348	70	739	58
	No	149	30	536	42
	Crude OR (95% CI) ^a^	1.69 *** (1.36–2.12)		Ref.	
	Adjusted OR (95% CI) ^a,b^	1.38 * (1.05–1.82)		Ref.	

Note: ^a^ Logistic regression. ^b^ Adjusted for age, gender, race, hypertension, ear infection, and diabetes. * *p* ≤ 0.05, *** *p* ≤ 0.001.

**Table 3:** Prevalence, odds ratios, and 95% confidence interval for low-frequency hearing loss, high-frequency hearing loss, and hearing loss, stratified by the numbers of illegal drug use.

Variables		Illegal Drugs Users				Nonusers (*n* = 1275)	
		Used 1 Type of Illegal Drug (*n* = 287)		Used ≥ 2 Types of Illegal Drugs (*n* = 210)			
		No.	%	No.	%	No.	%
Low-Frequency Hearing Loss							
	Yes	74	25.8	64	30.5	324	25.4
	No	213	74.2	146	69.5	951	74.6
	Crude OR (95% CI) ^a^	1.02 (0.76–1.37)		1.29 (0.94–1.77)		Ref.	
	Adjusted OR (95% CI) ^a,b^	0.81 (0.58–1.13)		1.06 (0.73–1.54)		Ref.	
High-Frequency Hearing Loss							
	Yes	190	66.2	151	71.9	720	56.5
	No	97	33.8	59	28.1	555	43.5
	Crude OR (95% CI) ^a^	1.51 ** (1.15–1.98)		1.97 *** (1.43–2.72)		Ref.	
	Adjusted OR (95% CI) ^a,b^	1.18 (0.84–1.65)		1.57 * (1.06–2.32)		Ref.	
Overall Hearing Loss							
	Yes	195	67.9	153	72.9	739	58
	No	92	32.1	57	27.1	536	42
	Crude OR (95% CI) ^a^	1.54 ** (1.17–2.02)		1.95 *** (1.41–2.69)		Ref.	
	Adjusted OR (95% CI) ^a,b^	1.26 (0.90–1.76)		1.60 * (1.08–2.37)		Ref.	

Note: ^a^ Logistic regression. ^b^ Adjusted for age, gender, race, hypertension, ear infection, and diabetes. * *p* ≤ 0.05, ** *p* ≤ 0.01, *** *p* ≤ 0.001.

**Table 4:** Prevalence, odds ratios, and 95% confidence interval for low-frequency hearing loss, high-frequency hearing loss, and hearing loss, stratified by the type of illegal drug use.

Variables		Illegal Drugs Users						Nonusers (*n* = 1275)	
		Ever Used Cocaine (*n* = 472)		Ever Used Heroin (*n* = 56)		Ever Used Methamphetamine (*n* = 209)			
		No.	%	No.	%	No.	%	No.	%
Low-Frequency Hearing Loss									
	Yes	132	28	21	37.5	59	28.2	324	25.4
	No	340	72	35	62.5	150	71.8	951	74.6
	Crude OR (95% CI) ^a^	1.14 (0.90–1.45)		1.76 * (1.01–3.07)		1.16 (0.83–1.60)		Ref.	
	Adjusted OR (95% CI) ^a,b^	0.88 (0.67–1.17)		1.03 (0.53–2.02)		1.08 (0.74–1.57)		Ref.	
High-Frequency Hearing Loss									
	Yes	328	69.5	45	80.4	142	67.9	720	56.5
	No	144	30.5	11	19.6	67	32.1	555	43.5
	Crude OR (95% CI) ^a^	1.76 *** (1.40–2.20)		3.15 *** (1.62–6.15)		1.63 ** (1.20–2.23)		Ref.	
	Adjusted OR (95% CI) ^a,b^	1.34 * (1.01–1.77)		1.91 (0.87–4.19)		1.43 (0.98–2.11)		Ref.	
Overall Hearing Loss									
	Yes	333	70.6	46	82.1	146	69.9	739	58
	No	139	29.4	10	17.9	63	30.1	536	42
	Crude OR (95% CI) ^a^	1.74 *** (1.39–2.18)		3.33 *** (1.67–6.67)		1.68 *** (1.23–2.31)		Ref.	
	Adjusted OR (95% CI) ^a,b^	1.38 * (1.04–1.82)		2.17 (0.97–4.84)		1.54 * (1.05–2.27)		Ref.	

Note: ^a^ Logistic regression. ^b^ Adjusted for age, gender, race, hypertension, ear infection, and diabetes. * *p* ≤ 0.05, ** *p* ≤ 0.01, *** *p* ≤ 0.001.

## Data Availability

Publicly available datasets were analyzed in this study. This data can be found here: https://www.cdc.gov/nchs/nhanes/index.htm (accessed on 12 November 2021).
